# Results of a nationwide survey on Japanese clinical practice in breast-conserving radiotherapy for breast cancer

**DOI:** 10.1093/jrr/rry095

**Published:** 2018-11-21

**Authors:** Norihiro Aibe, Kumiko Karasawa, Masahiko Aoki, Keiko Akahane, Yasuhiro Ogawa, Etsuyo Ogo, Shuichi Kanamori, Jiro Kawamori, Anneyuko I Saito, Kenshiro Shiraishi, Hiroshi Sekine, Seiji Tachiiri, Michio Yoshimura, Chikako Yamauchi

**Affiliations:** 1 Breast Cancer Group of the Japanese Radiation Oncology Study Group; 2 Department of Radiology, Kyoto Prefectural University of Medicine, 465 Kajii-cho, Kawaramachi-Hirokoji, Kamigyo-ku, Kyoto, Japan; 3 Department of Radiation Oncology, Tokyo Women's Medical University, School of Medicine, 8-1, Kawada-cho, Shinjuku-ku, Tokyo, Japan; 4 Department of Radiology and Radiation Oncology, Hirosaki University Graduate School of Medicine, 5 Zaifu, Hirosaki city, Aomori, Japan; 5 Department of Radiology, Saitama Medical Center Jichi Medical University, 1-847 Amanuma, Omiya, Saitama City, Saitama, Japan; 6 Director, Hyogo Prefectural Kakogawa Medical Center, 203 Kann, Kakogawa cityi, Hyogo, Japan; 7 Department of Radiology, Kurume University School of Medicine, 67 Asahi-Machi, Kurume City, Fukuoka, Japan; 8 Department of Radiation Oncology, Kindai University, Faculty of Medicine, 377-2 Ohno-Higashi, Osaka-Sayama City, Osaka, Japan; 9 Department of Radiation Oncology, St Luke`s International Hospital, 9-1 Akashi-cho, Chuo-ku, Tokyo, Japan; 10 Division of Radiation Oncology, Department of Radiology, Juntendo University School of Medicine, 3-1-3 Hongo, Bunkyo-ku, Tokyo, Japan; 11 Department of Radiology, Teikyo University, 2-11-1 Kaga, Itabashi-ku, Tokyo, Japan; 12 Department of Radiology, the Jikei University, School of Medicine, 3-25-8, Nishi-Shimbashi, Minato-ku,Tokyo, Japan; 13 Department of Therapeutic Radiology, Uji Tokushukai Medical Center, 145 Ishibashi, Maxima-cho, Uji City, Kyoto, Japan; 14 Department of Radiation Oncology and Image-applied Therapy, Kyoto University, Graduate School of Medicine, Yoshida-Konoe-cho, Sakyo-ku, Kyoto, Japan; 15 Department of Radiation Oncology, Shiga General Hospital, 30-4-5 Moriyama, Moriyama City, Shiga, Japan

**Keywords:** nationwide questionnaire survey, Japanese clinical practice, postoperative radiotherapy, breast-conserving treatment

## Abstract

The Breast Cancer Group of the Japanese Radiation Oncology Study Group conducted a nationwide questionnaire survey on the clinical practice of postoperative radiotherapy for breast-conserving treatment for breast cancer. This questionnaire consisted of 18 questions pertaining to the annual number of treated patients, planning method, contouring structure, field design, dose-fractionated regimen, application of hypofractionated radiotherapy, boost irradiation, radiotherapy for synchronously bilateral breast cancer, and accelerated partial breast irradiation. The web-based questionnaire had responses from 293 Japanese hospitals. The results indicated the following: treatment planning is performed using relatively similar field designs and delivery methods; the field-in-field technique is used at more than one-third of institutes; the commonest criteria for boost irradiation is based on the surgical margin width (≤5 mm) and the second commonest criteria was age (≤40 or ≤50 years), although some facilities applied a different age criterion (>70 years) for omitting a tumor bed boost; for conventional fractionation, almost all institutes delivered 50 Gy in 25 fractions to the conserved whole breast and 10 Gy in 5 fractions to the tumor bed. This survey revealed that 43% of hospitals offered hypofractionated radiotherapy, and the most common regimens were 42.56 Gy in 16 fractions for whole-breast irradiation and 10.64 Gy in 4 fractions for boost irradiation. Almost all of the facilities irradiated both breasts simultaneously for synchronously bilateral breast cancer, and accelerated partial breast irradiation was rarely offered in Japan. This survey provided an overview of the current clinical practice **of** radiotherapy for breast-conserving treatment of breast cancer in Japan.

## INTRODUCTION

For patients with early-stage breast cancer, radiotherapy to the conserved breast after breast-conserving surgery (post-BCS RT) reduces the risk of local recurrence and cancer-related death, with a potential survival benefit [1, 2]. For these reasons, post-BCS RT has gained widespread use for patients with early-stage breast cancer. Technological advances in radiation therapy and a deeper understanding of clinicopathological features of breast cancer have led to an increase in the variety of methods available for irradiating the conserved breast.

Currently, different options for radiotherapy are available for breast-conserving treatment. An increase in dose delivery methods has resulted from advanced techniques such as 3D conformal radiotherapy (3DCRT), the field-in-field technique (FIF) and intensity-modulated radiation therapy (IMRT). Some studies have shown that boost irradiation (BI) to the tumor bed brings better local control, especially in younger patients, although different clinical trials have used different dose-fraction regimens [3, 4]. Such evidence may result in the different adaptability and dose-fractionation regimens for BI among different hospitals. Furthermore, hypofractionated radiation schedules have been offered more frequently based on the high levels of evidence for long-term safety and efficacy [5, 6]. A greater understanding of breast cancer biology would allow for optimizing the field design for post-BCS RT. Several studies have reported that accelerated partial breast irradiation (APBI) or partial breast irradiation (PBI) provides satisfactory outcomes in some categories of early-stage breast cancer [7, 8]. It is thus possible that, based on such reports, diverse radiotherapy (dose–fraction regimens and targeted volumes) may be prevalent in clinical practice.

After the American College of Surgeons Oncology Group (ACOSOG) Z-0011 trial revealed the feasibility of milder surgical axillary management for patients with low-volume axillary disease, axillary dissection is being increasingly omitted in eligible patients (one or two positive sentinel lymph nodes, having undergone breast conservation, scheduled to receive postoperative radiotherapy and systemic adjuvant therapy) [9]. This landmark trial has altered the treatment paradigm for axillary management and has led to the recommendation to omit additional axillary surgery beyond sentinel lymph node biopsy in women meeting the ACOSOG Z0011 inclusion criteria by the National Comprehensive Cancer Network and the American Society of Clinical Oncology [10, 11]. However, the optimal radiation field for use in such patients with low-volume axillary disease treated by mild axillary management is still unclear, although a standard ‘tangent’ or a ‘high tangent’ is generally considered to be appropriate for axillary disease control. Such changes may lead to differences in targeted volumes for post-BCS RT.

Elucidating the details of the variation among hospitals in Japan may help improve medical practices and patient management, and bring to light new obstacles that need to be addressed. This information could also be used for future clinical trials and for helping to inform national health insurance policy. To assess whether variations exist in post-BCS RT practices among different Japanese institutions, the Breast Cancer Group of the Japanese Radiation Oncology Study Group (JROSG-BCG), with the support of the Japanese Society for Radiation Oncology (JASTRO), conducted a nationwide questionnaire survey on the clinical practice of post-BCS RT in Japan. The aim of this study was to report the results of the tendencies in the clinical practice of post-BCS RT in Japan from the nationwide survey.

## MATERIALS AND METHODS

The JASTRO approved and supported the initiative to conduct the questionnaire survey. This survey was conducted with institutional ethical approval. The JROSG-BCG formulated a web-based questionnaire system and requested the participation of all Japanese institutes with ≥1 full-time radiation oncologist and with an annual record of treating ≥10 patients with post-BCS RT in clinical practice. Radiation oncologists were requested to answer 18 questions related to post-BCS RT in six categories as follows (see **Supplementary Data 1** for details of the questionnaire): (i) the number of the patients treated annually (three questions); (ii) the planning method/the contouring structure (six questions); (iii) the field design (one question); (iv) the dose-fractionated regimen/the practice of hypofractionated radiotherapy (four questions); (v) the criteria for applying BI (two questions); and (vi) the practice of bilateral post-BCS RT/APBI (two questions). Open access to the questionnaire was available during April, May, July and August of 2016, for a total of 4 months. For convenience, university hospitals and hospitals designated for cancer treatment were termed specialized hospitals (SHs) since they specialized in cancer treatment, whereas the other hospitals were termed general hospitals (GHs).

## RESULTS

Completed questionnaires were received from 293 institutes. According to the statistical data of 2015 provided by the JASTRO Database Committee, the collection rate of this survey accounted for 55% (293/535) of the total number of institutes (535) that the JASTRO has authorized as facilities specializing in radiotherapy with ≥1 full-time radiation oncologist. Although 66% (192/293) of the institutes were GHs, we obtained responses from 89% (101/113) of SHs.

### Number of patients treated annually

**Fig. 1.**
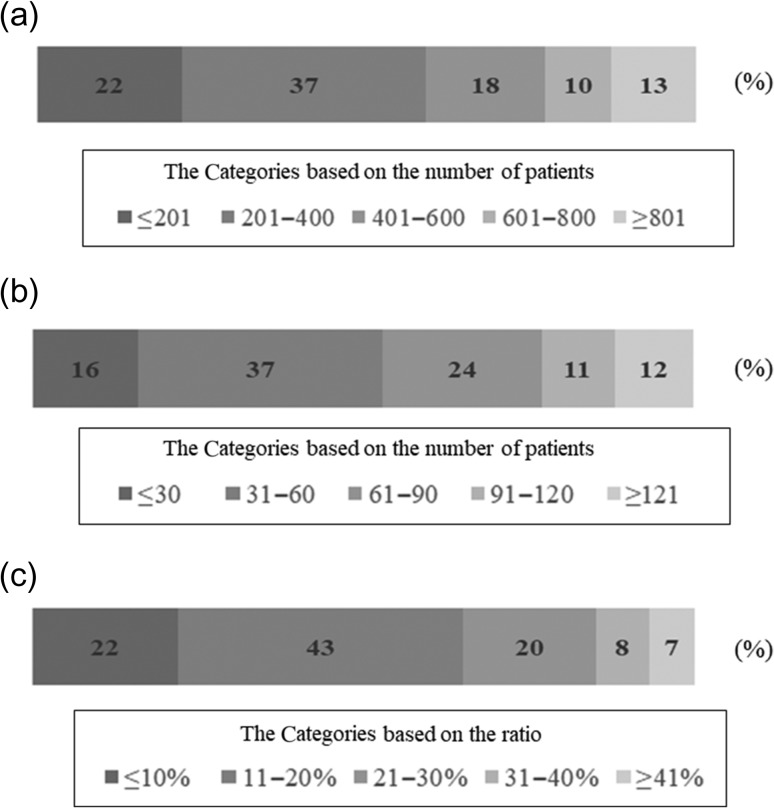
The percentage of hospitals categorized according to the number of the patients treated annually. (*a*) The percentage of hospitals categorized according to the total number of patients treated with RT annually. (*b*) The percentage of hospitals categorized according to the total number of patients treated with post-BCS RT annually. (*c*). The percentage of hospitals categorized according to the ratio of the annual number of patients treated with post-BCS RT to that of the annual number of patients treated with RT. RT = radiation therapy; post-BCS RT = radiotherapy after breast-conserving surgery.

Figure 1 shows the number and ratio of patients treated annually with RT or post-BCS RT. In this study, 77% (225/293) of hospitals conducted RT for ≤600 patients; 76% (224/293) of the included facilities conducted post-BCS RT for ≤90 women.

### Treatment planning/Contouring for treatment planning

**Fig. 2.**
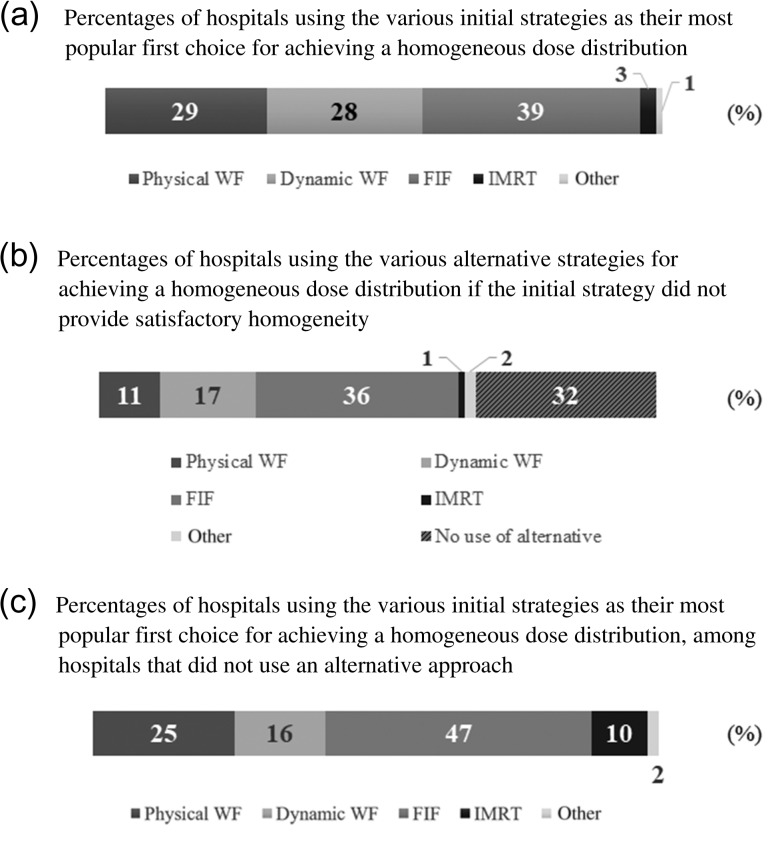
The percentage of hospitals categorized according to the methods employed to ensure homogeneity in dose distribution. (*a*). Percentages of hospitals using the various initial strategies as their most popular first choice for achieving a homogeneous dose distribution. (*b*) Percentages of hospitals using the various alternative strategies for achieving a homogeneous dose distribution if the initial strategy did not provide satisfactory homogeneity. (*c*) Percentages of hospitals using the various initial strategies as their most popular first choice for achieving a homogeneous dose distribution, among hospitals that did not use an alternative approach. WF = wedge filter; FIF = field-in-field technique; IMRT = intensity-modulated radiotherapy.

Computed tomography (CT)-based 3DCRT was performed at 98% (288/293) of the included hospitals. In 92% (271/293) of all hospitals, treatment plans were calculated with inhomogeneity correction and with the use of accurate and novel algorithms such as the superposition algorithm. To make homogenous dose distributions, 57% (167/293) of hospitals used physical or dynamic wedge filters and 39% (114/293) employed the FIF technique as the first approach used commonly in each hospital (Fig. 2). The FIF technique was the most commonly used alternative method to homogenize dose distributions (36%, 106/293), if the first approach failed to do it. Among the institutes where no alternative method was generally employed, 47% (45/95) of them used the FIF technique as the first approach. For contouring of the target and the organs at risk (OARs), 66% (192/293) of all institutes formulated a contour of the converted breast for the clinical target volume (CTV), while 66% (192/293) and 76% (222/293) of all institutes delineated the regions of interest such as the heart and the lung to evaluate the dose distributions, respectively.

### Field design of post-breast-conserving surgery radiotherapy

The questionnaire contained questions about the most popular landmarks selected to define the edges of a treatment field for post-BCS RT in each hospital (see **Supplementary data 2** for details of the survey result, **Figs S1–S6**). The middle line was the most common medial edge of the field (71%, 209/293). For the lateral edge, 50% (146/293) applied the boundary from the middle axillary line to posterior, though 4% (11/293) employed the more posterior landmarks such as the posterior axillary line or the anterior aspect of the ipsilateral latissimus. For the cranial edge, the sternal notch was selected by 55% (162/293) of hospitals. For the caudal edge, 64% (188/293) of facilities used the line 1–2 cm below the inframammary fold. The line ≥1 cm anterior to an ipsilateral nipple was the most commonly selected anterior edge for 86% (251/293) of all included institutions, while the posterior edges were based on the central lung distance (CLD) in 69% (202/293) of hospitals; this was the distance between the anterior edge of the lung and the midpoint of the posterior field edge. For the posterior edge, 25% (73/293) of institutes selected a CLD of <2 cm, 33% (96/293) selected a CLD of <2.5 cm, and 11% (33/293) selected a CLD of <3 cm.

### Dose and fractionation; criteria for hypofractionated whole-breast irradiation

**Fig. 3.**
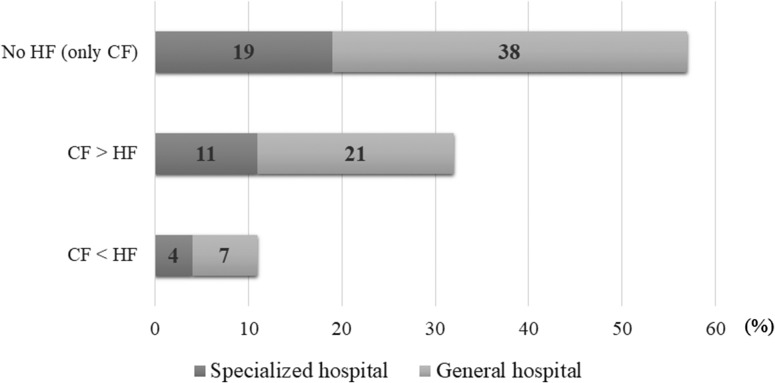
Dose fractionation: conventional fractionation vs hypofractionation. CF = conventional fractionation; HF = hypofractionation. In this investigation, specialized hospitals were defined as university hospitals or hospitals designated for cancer treatment; the remaining hospitals were termed ‘general hospitals’.

All participating facilities answered the question about the use of conventional fractionated (CF) and hypofractionated (HF) regimens, but we did not receive the detailed information on the HF dose fractionation from two facilities. Table 1A shows the total dose, dose per fraction, and the total fractions in CF post-BCS RT (dose in fraction <2.5 Gy). With the exception of four institutes that did not employ the CF regimen, 98% (284/289) of all hospitals used a protocol of 50 Gy in 25 fractions for whole-breast irradiation (WBI) and 92% (266/289) used a BI of 10 Gy in 5 fractions. Table 1B describes the variety of doses and fractionations of HF post-BCS RT (dose in fraction ≥2.5 Gy). Compared with CF regimens, there was a wider dispersion in HF schedules among different institutes, especially in those for the dose fractionations of BI. A total of 38% (111/293) of the hospitals employed HF regimens for WBI of 40–43.2 Gy in 16 fractions; the protocol of 42.56 Gy in 16 fractions was used in 30% (89/293) of hospitals and was the most common of all HF regimens. Eleven hospitals did not provide BI in HF schedules. In this study, 43% (127/293) of all hospitals conducted HF post-BCS RT and 11% (33/293) of facilities performed HF post-BCS RT more frequently in clinical practice than CF post-BCS RT (Fig. 3). Regarding the eligibility criteria for HF post-BCS RT, 40% (51/127) of the institutes employed the criteria recommended by the Japanese Breast Cancer Society (JBCS) [12]. The criteria included the following: (i) aged ≥50 years at diagnosis; (ii) pathologic stage of T1–2 N0 and receipt of breast-conserving surgery; (iii) no history of systemic chemotherapy; (iv) within the breast, the minimum dose was ≥93% and the maximum dose was ≤107% of the prescription dose.

**Table 1.** The list of doses and fractionations for whole-breast irradiation and boost irradiation, and the percentages of hospitals using this approach

	Total dose (Gy)	Does/Fr (Gy)	Total Fr	The number (%) of all hospitals	The number (%) of specialized hospitals
**A. Conventional fractionation**					
**WB**	50	2	25	286 (98)	101 (100)
	50.4	1.8	28	2 (0.7)	0 (0)
	47.5	2.375	20	1 (0.3)	0 (0)
	No regimen			4 (1)	0 (0)
	Total number			293	101
**Boost**	10	2	5	267 (92)	94 (93)
	9	3	3	8 (3)	3 (3)
	16	2	8	6 (2)	3 (3)
	4–15	2–3	2–7	8 (3)	1 (1)
	No regimen			4 (1)	0 (0)
	Total number			293	101
**B. Hypofractionation**					
**WB**	40.05	2.67	15	5 (2)	3 (3)
	40–43.2	2.5–2.7	16	111 (38)	41 (41)
	42.5–50	2.5	17–20	9 (3)	1 (1)
	No regimen			166 (57)	56 (56)
	Total number			291	101
**Boost**	5–9	2.5–3	3	23 (7.9)	2 (2)
	10–10.64	2.5–2.66	4	69 (23.7)	28 (28)
	10–15	2–2.66	5–6	22 (7.6)	9 (9)
	No regimen			177 (60.8)	62 (62)
	Total number			291	101

Fr = fractionation; WB = whole breast.

### Criteria to apply boost irradiation

This survey asked the participating institutes to list the eligibility criteria for offering BI to the tumor bed. All participating hospitals answered the question regarding the eligibility criteria, but in the analysis of surgical margin conditions, we used 89% (262/293) of answers regarding the superficial margin close to the skin or the deep margin close to the muscle and 95% (278/293) of answers regarding the lateral (horizontal) margin, because these answers were clear enough to analyze. Table 2 shows the factors of each institute taken into consideration in determining whether to use BI and the dominant factors that had a strong impact on the decision-making process. Surgical margin was the most commonly used determining factor. Overall, 97% (283/293) of hospitals considered surgical margin as an important factor for decision-making, and 94% (275/293) of the facilities regarded surgical margin as a dominant factor, although the definition of surgical margin as a determining factor differed between the hospitals in this survey (Table 3). For the criteria, some institutes used the margin width, whereas others referred to the margin status (‘close’ or ‘positive’) on the histological reports. As for the lateral margin, 70% (194/278) of the hospitals regarded a ≤5-mm margin width as a determinant for the choice of BI. For the superficial margin close to the skin or the deep margin close to the muscle, the most commonly used marginal determinant for BI was also the ≤5-mm margin width (45%, 119/262 or 43%, 113/262, respectively). Age was the second factor used commonly in the decision about BI. In 38% (110/293) of facilities, age was regarded as one of the deciding factors, and it was regarded as a dominant factor in 19% (55/293) of hospitals. According to the analysis of 97 answers reporting clear information on the thresholds of age for BI, the common thresholds were ≤40 and ≤50 years (36%, 35/97 and 52%, 50/97, respectively), but four facilities used age >70 years as the age range for omitting BI (see **Supplementary data 2**, **Table S1**).

**Table 2.** The list of important factors taken into consideration in making the decision to deliver boost irradiation, and the percentage of hospitals using this approach

Elements	Percentage of hospitals (%)	
	An important factor	A dominant factor*
Age	38	19
Surgical margin condition	97	94
Nodal metastasis	5	2
Lymphovascular invasion	8	0.3
Subtypes of breast cancer	6	2
Others	7	6

*A dominant factor is defined as a factor having a strong impact on the decision to apply BI, i.e. the use of BI could be determined solely by the dominant factor. BI = boost irradiation.

**Table 3.** The diversity of the eligibility criteria for choice of boost irradiation: percentage of hospitals arranged according to the margin width or margin status (in each direction)

Eligibility condition of surgical margin	Percentage of hospitals (%)		
	Superficial margin	Deep margin	Lateral margin
Margin width^a^ ≤1 mm	3	2	2
Margin width^a^ ≤2 mm	3	3	3
Margin width^a^ ≤5 mm	45	43	70
Margin width^a^ = 0 mm	26	29	5
Margin status (close margin)^b^	5	5	5
Margin status (positive margin)^b^	13	12	12
Others	4	5	2

^a^The ‘margin width’ was the distance between the area of the tumor invasion and the specimen edge.

^b^The ‘margin status’ (close margin or positive margin) was provided by the histological report (close margin or positive margin).

### Method for the bilateral post-BCS RT/practice of APBI

In patients with synchronously diagnosed bilateral breast cancers, 85% (250/293) of all hospitals delivered the post-BCS WBI to the bilateral conserved breast without any interval between irradiation to the right and the left sides. A total of 7% (21/293) of the hospitals conducted these procedures with an interval of a few weeks between procedures, 4% (13/293) of the facilities offered these procedures sequentially, and the other institutes used their institutional procedures. Only 2% (5/293) of the institutes offered APBI as part of clinical practice, and 1% (3/293) offered this procedure as part of clinical trials. In this study, 97% (284/293) of all hospitals in Japan did not offer APBI.

## DISCUSSION

As far as we know, this nationwide web-based questionnaire survey is the first investigation to reveal the Japanese clinical practice of post-BCS RT with an analysis of radiation planning. All of the 293 participating institutes responded, and the survey results contain the opinions of the majority of the SHs (89%, 101/113). Therefore, the results of this survey likely indicate the tendencies of current Japanese clinical practice in post-BCS RT.

Almost all hospitals conducted post-BCS RT using 3DCRT planning based on CT simulation with inhomogeneity correction and with relatively novel calculation algorithms (e.g. superposition). There were no major discrepancies in field designs between different institutions, although some minor differences were noted. The contours of the conserved breast, heart, and lung were delineated for precise dose delivery in more than half of the included institutes. This survey revealed the popularity of the FIF technique for achieving homogenous dose delivery. This result might be a reflection of the prevalence of 3DCRT in Japan, which might have helped increase awareness of the efficacy of the FIF technique among clinicians. The importance of ensuring homogeneity of dose delivery to the conserved breast has been widely recognized and will likely lead to a more widespread use of the FIF technique. Furthermore, the updated guideline of an ASTRO, which recommended 3D conformal treatment planning with the FIF technique, might promote the spread of the FIF technique in Japan [6].

This investigation revealed the uniformity in the schedules of CF post-BCS RT and the frequency of HF post-BCS RT use among Japanese institutes. CF schedules in almost all institutes used 50 Gy in 25 fractions (5 days per week) for WBI and 10 Gy in 5 fractions for BI. HF schedules were more varied than those of CF and differed in total doses and dose fractionations. The most common regimens were 42.56 Gy in 16 fractions (5 days per week) for WBI and 10.64 Gy in 4 fractions for BI. Our results showed that 43% of hospitals employed the HF regimen, and 11% offered the HF schedule more frequently than they did the CF schedule. In the UK, the HF regimen (40 Gy in 15 fractions) has been adopted widely based on the long-term follow-up results of randomized controlled trials [13]. Several studies have revealed the prevalence of the HF regimen in Canada and the USA [14, 15]. The HF regimen was not used commonly in Japan compared with European countries such as the UK or the Netherlands. One of the reasons might be that the results of the JCOG 0906, a Japanese multicenter safety trial of HF post-BCS RT [16], had not commenced at the start of this survey. We speculate that the HF regimen has likely gained (and will gain more) popularity in Japan, based on the increased awareness of its efficacy [17, 18], the future announcement of the JCOG0906 result, and the ASTRO’s updated guideline, which contained more flexible eligibility criteria for use of the HF regimen than the JBCS criteria [6].

According to this survey, surgical margin was the most common determining factor for selection of BI, and age was the second factor, although different criteria were adopted in different institutes. Of the Japanese institutes in this survey, 98% considered surgical margin as an important factor, and 94% regarded it as the dominant factor to be used solely in determining the selection of BI. Many of the facilities in this survey used the ≤5-mm margin width in determining the selection of BI. The EORTC 22881–10882 trial clearly revealed the benefit of BI in terms of local control in patients with microscopically complete excision of invasive disease, although BI could lead to worse cosmetic outcomes or severe fibrosis without an apparent impact on survival benefit [3, 4]. The Japanese tendency to base the use of BI on margin width might be related to the hypothesis that a narrower margin is associated with a risk of local recurrence and the concern that BI could cause a worse cosmetic outcome. However, the panel of the Society of Surgical Oncology and ASTRO concluded that choosing BI should not be dependent on the margin width [19]. Their meta-analysis showed that there was a weak relationship between local recurrence, margin width and dose in contemporary multimodality treatment, and concluded that in patients with negative margins (no ink on the tumor), the use and dose of BI should be based on an *a priori* estimation of local recurrence risk in the ipsilateral converted breast, and should not be determined, in isolation, by the width of the surgical margin. According to their report, to estimate the *a priori* risk of local recurrence, not only margin width but also the other factors (age, subtype, the use of systemic therapies, etc.) should be taken into account. This survey showed that some institutes regarded the other factors (age, subtype, etc.) as determining factors for the selection of BI, but (in 2016) many institutes seemed to put the emphasis on margin width in selecting BI in Japan. In the future, the use of all of the criteria related to the *a priori* risk of local recurrence is likely to spread in Japan, with novel and future evidence for the benefits of optimizing the balance between the benefit and burden of BI. The changes in the Japanese trends in the criteria for selecting BI are worthy topics for future studies.

In regard to age, 38% of Japanese institutes in this survey regarded age as an important factor and 19% regarded it as the dominant factor to use in selection of BI. Many institutes used the age range of ≤40 or ≤50 years as the criterion for applying BI, as young age is associated with local recurrence. The Oxford meta-analysis of breast-conserving surgery demonstrated an inverse relationship between the rate of any first recurrence and age [1]. The EORTC 22881–10882 trial showed that the relative benefit of BI for local control was larger in younger patients than in older patients, and the absolute risk reduction at 20 years was the largest in patients ≤40 years of age (36.0% in the no boost group versus 24.4% in the boost group) [3]. Japanese institutes seemed to use the age range of ≤40 or ≤50 years based on the above evidence.

A majority (85%) of the institutes included in the survey performed bilateral post-BCS RT synchronously in patients with synchronously diagnosed bilateral breast cancers, indicating the high prevalence of this procedure in Japan. This survey also revealed that most of the institutes (98%) did not perform APBI in clinical practice, and thus APBI may not be the method of choice for post-BCS RT in Japan. One of the reasons for this is likely to be that the Japanese Breast Cancer Society Clinical Practice Guideline for Breast Cancer had recommended APBI should be performed in a clinical trial [12] . It is also possible that this tendency may be due to the variation in the outcomes of APBI in different studies and the questionable superiority of APBI over WBI [20]. However, APBI or PBI could be available for selected patients in the near future, based on newly accumulating evidence [8, 21, 22]. Changes in the Japanese trends regarding the use of APBI and PBI would be worthy topics for future studies.

This survey has some limitations. First, this questionnaire was not answered by all eligible institutes in Japan (institutions with ≥1 radiation oncologist working full-time and with ≥10 patients treated with post-BCS RT annually), which implies that the results may not be representative of clinical practices of post-BCS RT in Japan. However, the majority of the SHs (which are the primary cancer treatment centers in Japan) participated in this survey. Therefore, we believe that this survey presents an accurate overview of the tendencies in the clinical practice of post-BCS RT in 2016 in Japan. Second, the respondents had to select the most appropriate answers from several options in this questionnaire, which may have unintentionally guided them. Third, this survey disclosed the landmarks of field edges for treatment planning, although this information might be insufficient for detecting precise differences in dose distributions on the conserved breasts as applied by the different institutes. Future investigations would need detailed digital imaging data to better understand the variation in prevalent radiation therapy.

The findings of this nationwide survey provided an overview of the current clinical practices of post-BCS RT in Japan and the results can be compared with those of future investigations into Japanese practice of post-BCS RT. This study has provided not only the features of current Japanese practice of post-BCS RT in 2016, but also topics for future studies.

## Supplementary Material

Supplementary Data

Supplementary Data

## References

[1] Early Breast Cancer Trialists’ Collaborative Group (EBCTCG), Darby S, McGale P Effect of radiotherapy after breast-conserving surgery on 10-year recurrence and 15-year breast cancer death: meta-analysis of individual patient data for 10,801 women in 17 randomised trials. Lancet 2011;378:1707–16. 22019144 10.1016/S0140-6736(11)61629-2 PMC3254252

[2] Clarke M, Collins R, Darby S et al Effects of radiotherapy and of differences in the extent of surgery for early breast cancer on local recurrence and 15-year survival: an overview of the randomised trials. Lancet 2005;366:2087–106. 16360786 10.1016/S0140-6736(05)67887-7

[3] Bartelink H, Maingon P, Poortmans P et al Whole-breast irradiation with or without a boost for patients treated with breast-conserving surgery for early breast cancer: 20-year follow-up of a randomised phase 3 trial. Lancet Oncol 2015;16:47–56. 25500422 10.1016/S1470-2045(14)71156-8

[4] Kindts I, Laenen A, Depuydt T et al Tumour bed boost radiotherapy for women after breast-conserving surgery. Cochrane Database Syst Rev 2017;11:CD011987. 29105051 10.1002/14651858.CD011987.pub2 PMC6486034

[5] Valle LF, Agarwal S, Bickel KE et al Hypofractionated whole breast radiotherapy in breast conservation for early-stage breast cancer: a systematic review and meta-analysis of randomized trials. Breast Cancer Res Treat 2017;162:409–17. 28160158 10.1007/s10549-017-4118-7

[6] Smith BD, Bellon JR, Blitzblau R et al Radiation therapy for the whole breast: executive summary of an American Society for Radiation Oncology (ASTRO) evidence-based guideline. Pract Radiat Oncol 2018;8:145–52. 29545124 10.1016/j.prro.2018.01.012

[7] Correa C, Harris EE, Leonardi MC et al Accelerated partial breast irradiation: executive summary for the update of an ASTRO evidence-based consensus statement. Pract Radiat Oncol 2017;7:73–9. 27866865 10.1016/j.prro.2016.09.007

[8] Coles CE, Griffin CL, Kirby AM et al Partial-breast radiotherapy after breast conservation surgery for patients with early breast cancer (UK IMPORT LOW trial): 5-year results from a multicentre, randomised, controlled, phase 3, non-inferiority trial. Lancet 2017;390:1048–60. 28779963 10.1016/S0140-6736(17)31145-5 PMC5594247

[9] Giuliano AE, Ballman KV, McCall L et al Effect of Axillary Dissection vs No Axillary Dissection on 10-Year Overall Survival among Women with Invasive Breast Cancer and Sentinel Node Metastasis: the ACOSOG Z0011 (Alliance) randomized clinical trial. JAMA 2017;318:918–26. 28898379 10.1001/jama.2017.11470 PMC5672806

[10] National Comprehensive Cancer Network *NCCN Guidelines Version I 2018, March 20, 2018. Breast Cancer* https://www.nccn.org/professionals/physician_gls/pdf/breast.pdf (24 September 2018, date last accessed).

[11] Lyman GH, Somerfield MR, Bosserman LD et al Sentinel lymph node biopsy for patients with early-stage breast cancer: American Society of Clinical Oncology Clinical Practice Guideline Update. J Clin Oncol 2017;35:561–4. 27937089 10.1200/JCO.2016.71.0947

[12] Yamauchi C, Sekiguchi K, Nishioka A et al The Japanese Breast Cancer Society Clinical Practice Guideline for radiation treatment of breast cancer, 2015 edition. Breast Cancer 2016;23:378–90. 26883534 10.1007/s12282-016-0672-9

[13] Haviland JS, Owen JR, Dewar JA et al The UK Standardisation of Breast Radiotherapy (START) trials of radiotherapy hypofractionation for treatment of early breast cancer: 10-year follow-up results of two randomised controlled trials. Lancet Oncol 2013;14:1086–94. 24055415 10.1016/S1470-2045(13)70386-3

[14] Ashworth A, Kong W, Whelan TA et al A population-based study of the fractionation of postlumpectomy breast radiation therapy. Int J Radiat Oncol Biol Phys 2013;86:51–7. 23433799 10.1016/j.ijrobp.2012.12.015

[15] Bekelman JE, Sylwestrzak G, Barron J et al Uptake and costs of hypofractionated vs conventional whole breast irradiation after breast conserving surgery in the United States, 2008–2013. JAMA 2014;312:2542–50. 25494006 10.1001/jama.2014.16616 PMC4271796

[16] Ishikura S, Ito Y, Hiraoka M JCOG Radiation Therapy Study Group: history and achievements. Jpn J Clin Oncol 2011;41:1241–3. 21980050 10.1093/jjco/hyr126

[17] Koulis TA, Phan T, Olivotto IA Hypofractionated whole breast radiotherapy: current perspectives. Breast Cancer 2015;7:363–70. 26604820 10.2147/BCTT.S81710 PMC4629948

[18] Kim KS, Shin KH, Choi N et al Hypofractionated whole breast irradiation: new standard in early breast cancer after breast-conserving surgery. Radiat Oncol J 2016;34:81–7. 27306774 10.3857/roj.2016.01697 PMC4938347

[19] Moran MS, Schnitt SJ, Giuliano AE et al Society of Surgical Oncology–American Society for Radiation Oncology consensus guideline on margins for breast-conserving surgery with whole-breast irradiation in stages I and II invasive breast cancer. J Clin Oncol 2014;32:1507–15. 24516019 10.1200/JCO.2013.53.3935

[20] Hickey BE, Lehman M, Francis DP et al Partial breast irradiation for early breast cancer. Cochrane Database Syst Rev 2016;7:CD007077. 27425375 10.1002/14651858.CD007077.pub3 PMC6457864

[21] Correa C, Harris EE, Leonardi MC et al Accelerated partial breast irradiation: executive summary for the update of an ASTRO evidence-based consensus statement. Pract Radiat Oncol 2017;7:73–9. 27866865 10.1016/j.prro.2016.09.007

[22] Polgár C, Ott OJ, Hildebrandt G et al Late side-effects and cosmetic results of accelerated partial breast irradiation with interstitial brachytherapy versus whole-breast irradiation after breast-conserving surgery for low-risk invasive and *in-situ* carcinoma of the female breast: 5-year results of a randomised, controlled, phase 3 trial. Lancet Oncol 2017;18:259–68. 28094198 10.1016/S1470-2045(17)30011-6

