# Supplementary material for: Results of a nationwide survey on Japanese clinical practice in breast-conserving radiotherapy for breast cancer
Source: J Radiat Res. 2018 Nov 21;60(1):142–9. doi: 10.1093/jrr/rry095 (PMC6373682; doi:10.1093/jrr/rry095)
Supplement: Supplementary Data [file rry095_supplementary_data_1_rev.docx]

1. For the number of patients annually treated with RT in your institute, which of the following ranges is true of your institute?

a. <100 b. 101–200 c. 201–400 d. 401-600 e. 601-800

f. 801-1000 g. >1000

2. For the number of patients annually treated for post-BCS WBI in your institute, which of the following ranges is true of your institute?

a. <30 b. 31-60 c. 61-90 d. 91-120 e. 121-150 f. 151-200

g. 201-400 h. 401-600 i. 601-800 j. >800

3. For the ratio of the number of patients annually treated with post-BCS WBI to those treated with RT in your institute, which of the following ranges is true of your institute?

a. <5% b. 5-10% c. 11-20% d. 21-30% e. 31-40%

f. 41-50% g. 51-60% h. 61-70% i. 71-80% j. >80%

4. Answer the following questions about post-BCS WBI.

4-1. Which of the following is true of your institute?

1. We do not undergo post-BCS WBI with the hypofractionated schedule for those whose dose per fraction is 2.5 Gy or more.
2. We undergo the post-BCS WBI more commonly with the conventional schedule, for those whose dose per fraction is <2.5 Gy.
3. We undergo the post-BCS WBI more commonly with the hypofractionated schedule than the conventional.

4-2. What dose do you generally deliver to the conserved breast for post-BCS WBI or tumor bed for BI with the conventional schedule, whose dose per fraction is <2.5 Gy, in your institute?

a. Whole breast irradiation Dose per fraction ______ Gy, Total dose ______ Gy

b. Boost irradiation Dose per fraction ______ Gy, Total dose ______Gy

4-3. What dose do you generally deliver to the conserved breast for post-BCS WBI or tumor bed for BI with the hypofractionated schedule, whose dose per fraction is 2.5 Gy or more, in your institute?

a. Whole breast irradiation Dose per fraction ______ Gy, Total dose ______ Gy

b. Boost irradiation Dose per fraction ______ Gy, Total dose ______Gy

4-4. Choose the Radiotherapy Treatment Planning System and dose-calculation algorithm used in your institute for the planning of post-BCS WBI.

- Radiotherapy Treatment Planning System: Which of the following systems is used in your institute?

a. Eclipse b. XiO c. Pinnacle d. other ( )

- Dose-calculation algorithm 1: Which of the following algorithms is used in your institute?

a. PBC b. AAA c. Convolution d. Superposition e. Acurous XB

f. Monte Carlo g. other ( )

- Dose-calculation algorithm 2: Is the heterogeneity collection used to make a treatment plan in your institute?

a. Yes, it is. b. No, it is not.

4-5. Which method is generally used to make a treatment plan in your institute?

1. 2-dimensional radiotherapy planning
2. 3-dimensional radiotherapy planning
3. other ( )

4-6. Which of the following methods is used in your institute to make a homogenous dose distribution?

1. Which is the most popular method?

a. Physical wedge filter b. Dynamic wedge filter c. Field-in-field method

d. IMRT e. other ( )

2. Which method will be used as the alternative one when the most popular method does not make a homogenous delivery?

a. Physical wedge filter b. Dynamic wedge filter c. Field-in-field method

d. IMRT e. We never use the alternative method.

f. other ( )

4-7. Choose the appropriate options to the following questions regarding BI.

1. Which factors are used to make the decision to offer BI? (multiple answers allowed)

a. age b. the condition of surgical margin c. the status of the nodal involvement

d. the status of the lymphovascular invasion e. subtype

f. other ( )

2. Which are the dominant factors for offering BI? **A dominant factor is defined as a factor having a strong impact on the decision to apply BI, so that the use of BI could be determined solely by the dominant factor.** (multiple answers allowed)

a. age

If you choose “age”, list your criteria for it.

b. the condition of surgical margin

If you choose “the condition of the surgical margin”, list your criteria for it.

c. the status of the nodal involvement

If you choose “the status of the nodal involvement”, list your criteria for it.

d. the status of the lymphovascular invasion

If you choose “the status of the lymphovascular invasion”, list your criteria for it.

e. subtype

If you choose “subtype”, list your criteria for it.

f. other ( )

4-8. Answer the following question when the hypofractionated schedule is used in your institute.

Which of the following is true of your policy for hypofractionated post-BCS WBI?

1. All candidates for post-BCS WBI undertake hypofractionated RT.
2. The candidates for post-BCS WBI, who do not need regional nodal irradiation, undertake the hypofractionated RT.
3. The hypofractionated RT is administered based on the requests of a patient or surgeon when the patient meets the conditions recommended by the ASTRO or JBCS.
4. The hypofractionated RT is administered based on the requests of a patient or surgeon even if the patient does not meet the conditions recommended by the ASTRO or JBCS.
5. The hypofractionated RT is administered based on our own criteria.

Please list the criteria if you choose “e”.

4-9. Choose an appropriate option to the following question about the post-BCS WBI for a patient with synchronous bilateral breast cancer.

1. The post-BCS WBIs to the bilateral conserved breasts are administered synchronously without any interval.
2. The post-BCS WBIs to the bilateral conserved breasts are administered synchronously with some interval.
3. The post-BCS WBIs to the bilateral conserved breasts are administered sequentially. One side of the breast is first irradiated and then the other one.
4. Another method is used in our institute.

If you choose “d”, please mention your method.

4-10. Select an appropriate choice for the following question about the APBI

1. Our institute does not offer the APBI
2. Our institute offers the APBI based on the conditions of the patients who have undergone breast-conserving surgery.

If you choose “b”, please mention the criteria of the APBI and method to deliver the APBI

5. Answer the following questions about the contouring for treatment planning.

5-1. Pick the most appropriate answer about the contouring of the conserved breast.

1. We delineate the contour of the conserved breast based on the breast cancer atlas of the Radiation Therapy Oncology Group (1).
2. We delineate the contour of the conserved breast based on our own policy.
3. We do not delineate the contour of the conserved breast but draw the contour of the alternative structure for treatment planning.
4. We do not need the structure to delineate for treatment planning.
5. other.

If you choose “e”, please mention the approach used at your institute.

5-2. Select the most appropriate answer about the contouring of the heart and the evaluation of the dose to the heart for treatment planning.

1. We delineate the contour of heart and evaluate the dose of the heart, modifying the field design accordingly based on the dose of the heart.
2. Although we draw the contouring of the heart and evaluate the dose of the heart, we rarely modify the field design accordingly based on the dose of the heart.
3. We usually make a field design without any consideration of the dose to heart.
4. other

If you choose “d”, please mention your own policy.

5-3. Select the most appropriate answer about the contouring of the lung and the evaluation of the dose to the lung for treatment planning.

1. We delineate the contour of the lung and evaluated the dose of the lung, modifying the field design accordingly based on the dose of the lung.
2. Although we draw the contouring of the lung and evaluate the dose of the lung, we rarely modify the field design accordingly based on the dose of the lung.
3. We usually make a field design without any consideration of the dose to the lung.
4. other

5-4. Identify the landmark structure of each 6-direction field edge (medial, lateral, cranial, caudal, anterior, and posterior).

- Medial edge

a. the middle markers put on the skin when a simulation CT was obtained.

b. the middle line of the body

c. the edge line dependent on the PTV

d. other

- Lateral edge

a. the lateral markers put on the skin when a simulation CT was obtained.

b. the middle axillary line

c. the line between the middle and posterior axillary lines

d. the posterior axillary line

e. the line anterior to the latissimus dorsi

f. the edge line dependent on the PTV

g. other

- Cranial edge

a. the cranial markers put on the skin when a simulation CT was obtained.

b. the line cranial to the sternal notch

c. the line cranial to the sternal attachment of the secondary rib

d. the edge line dependent on the PTV

e. other

- Caudal edge

a. the caudal markers put on the skin when a simulation CT was obtained.

b. the line 1-2 cm below the inframammary fold

c. the edge line dependent on the PTV

d. other

- Anterior edge

a. the line 1-2 cm anterior to the surface of the ipsilateral nipple

b. the line >2 cm anterior to the surface of the ipsilateral nipple

c. the edge line dependent on the PTV

d. other

- Posterior edge

a. the CLD ≤2 cm

b. the CLD ≤2.5 cm

c. the CLD ≤3.0 cm

d. the edge line dependent on the PTV

e. other

< Abbreviation List >

RT, radiotherapy; post-BCS WBI, whole breast irradiation after breast-conserving surgery; BI, boost irradiation; IMRT, intensity-modulated radiation therapy; ASTRO, **American Society for Radiation Oncology**; JBCS, **Japanese Breast Cancer Society**; APBI, accelerated partial breast irradiation; PTV, planning target volume; CLD, central lung distance

< Reference >

1. Radiation Therapy Oncology Group (RTOG). Breast cancer atlas for radiation therapy planning. <https://www.rtog.org/LinkClick.aspx?fileticket=vzJFhPaBipE>= (27 November 2017, date last accessed)
