# Supplementary material for: Results of a nationwide survey on Japanese clinical practice in breast-conserving radiotherapy for breast cancer
Source: J Radiat Res. 2018 Nov 21;60(1):142–9. doi: 10.1093/jrr/rry095 (PMC6373682; doi:10.1093/jrr/rry095)
Supplement: Supplementary Data [file rry095_supplementary_data_2_rev.docx]

**Supplementary data 2: Figure S1-S6 and Table S1**

- **Figure S1. The variety of medial edge lines of the field used by different institutes (%).**

**
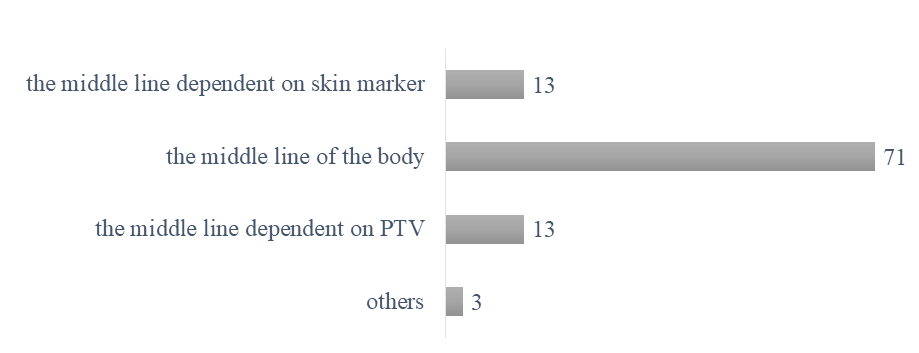
**

- **Figure S2. The variety of lateral edge lines of the field used by different institutes (%).**

**
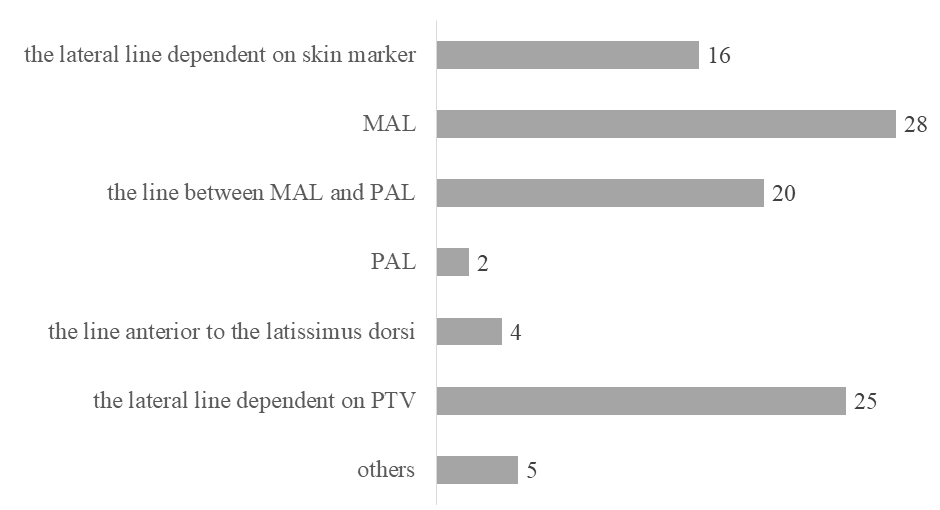
**

MAL, middle axillary line; PAL, posterior axillary line

- **Figure S3. The variety of cranial edge lines of the field used by different institutes (%).**

**
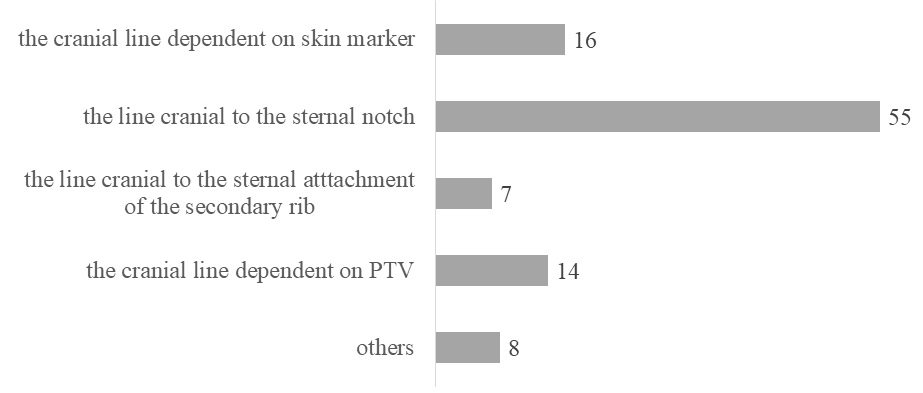
**

- **Figure S4. The variety of caudal edge lines of the field used by different institutes (%).**

**
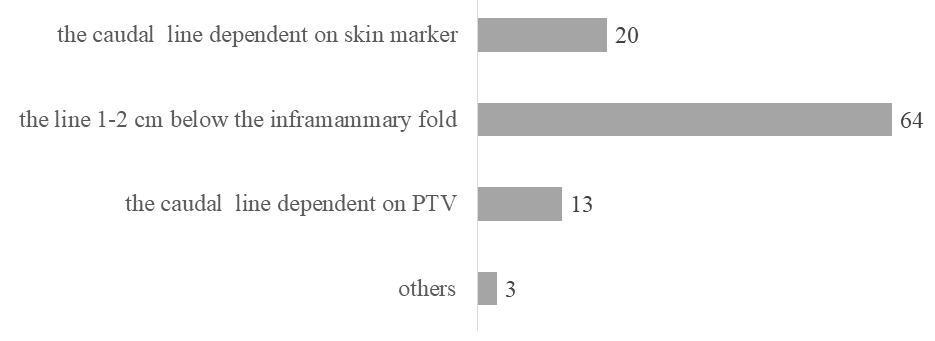
**

- **Figure S5. The variety of anterior edge lines of the field used by different institutes (%).**

**
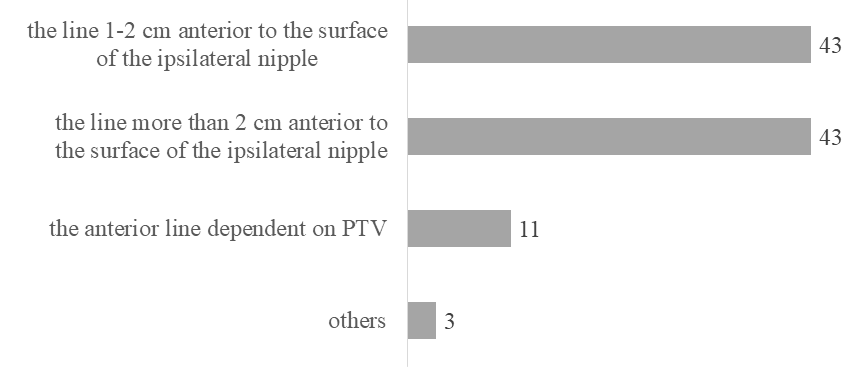
**

- **Figure S6. The variety of posterior edge lines of the field used by different institutes (%).**

**
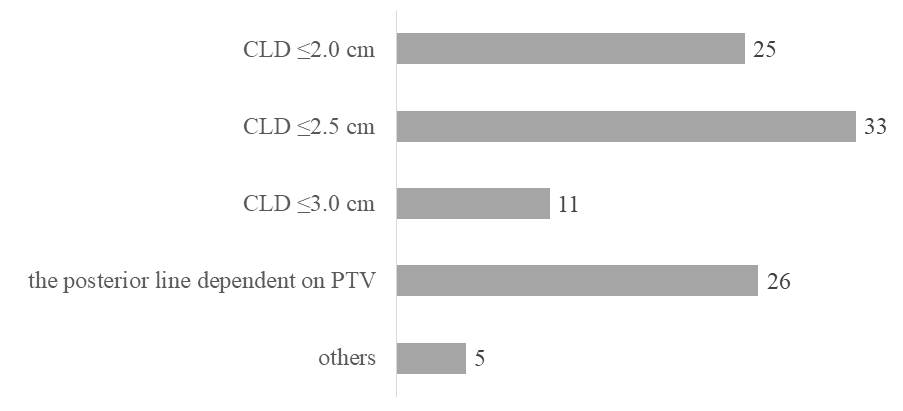
**

CLD, central lung distance

- **Table S1. The threshold of age for offering or omitting boost irradiation: the number of hospitals using the threshold of age as a factor to determine the choice of the boost irradiation in combination with the other risk factors.**

| **The threshold age for offering (years)** | **The number of the hospitals**  **using the threshold** |
| --- | --- |
| **≤35** | **3** |
| **≤40** | **35** |
| **≤45** | **8** |
| **≤50** | **50** |
| **≤60** | **1** |
| **The threshold age for omitting (years)** | **The number of the hospitals**  **using the threshold** |
| **>70** | **2** |
| **>75** | **1** |
| **>80** | **3** |
| **Others** | **2** |

**.**
